# Development of a protocol for whole-lung *in vivo* lung perfusion-assisted photodynamic therapy using a porcine model

**DOI:** 10.1117/1.JBO.29.11.118001

**Published:** 2024-11-15

**Authors:** Khaled Ramadan, Tina Saeidi, Edson Brambate, Vanderlei Bagnato, Marcelo Cypel, Lothar Lilge

**Affiliations:** a University of Toronto, Faculty of Medicine, Department of Surgery, Toronto, Ontario, Canada; b University of Toronto, Faculty of Medicine, Department of Medical Biophysics, Toronto, Ontario, Canada; c Toronto General Hospital, University Health Network, Toronto, Ontario, Canada; d Texas A and M University, Department of Biomedical Engineering, College Station, Texas, United States; e University of Sao Paulo, Institute of Physics of São Carlos, São Paulo, Brazil; f University Health Network, Princess Margaret Cancer Centre, Toronto, Ontario, Canada

**Keywords:** photodynamic therapy, full Monte, lung perfusion, dosimetry, lung cancer

## Abstract

**Significance:**

Standard treatments for isolated lung metastases remain a clinical challenge. *In vivo* lung perfusion technique provides flexibility to overcome the limitations of photodynamic therapy (PDT) by replacing the blood with acellular perfusate, allowing greater light penetration.

**Aim:**

Using Monte Carlo-based simulations, we will evaluate the abilities of a light delivery system to irradiate the lung homogenously. Afterward, we aim to demonstrate the feasibility and safety profile of a whole-lung perfusion-assisted PDT protocol using 5-ALA and Chlorin e6.

**Approach:**

A porcine model of a simplified lung perfusion procedure was used. PDT was performed at 630 or 660 nm with 5-ALA or Chlorin e6, respectively. Light fluence rate measurements and computed tomography (CT) scan segmentations were used to create *in silico* models of light propagation. Physiologic, gross, CT, and histological assessment of lung toxicity was performed 72 h post-PDT.

**Results:**

Dose-volume histograms showed homogeneity of light intensity throughout the lung. Predicted and measured fluence rates showed strong reliability. The photodynamic threshold of 5-ALA was $2.10\times 10^{17}\pm 8.24\times 10^{16}\;h\nu /{\mathrm{cm}}^{3}$, whereas Chlorin e6 showed negligible uptake in lung tissue.

**Conclusions:**

We lay the groundwork for personalized preoperative *in silico* dosimetry planning to achieve desired treatment volumes within the therapeutic range. Chlorin e6 demonstrated the greatest therapeutic potential, with a minimal uptake in healthy lung tissues.

## 1 Introduction

The lungs are a significant reservoir of metastatic burden, with isolated pulmonary metastases frequently affecting patients with sarcomas and colorectal carcinomas.^1^ Approximately, 20% of sarcoma patients and 10% to 25% of colorectal cancer patients will develop isolated pulmonary metastases.^2^^,^^3^ To date, surgical metastasectomy is the mainstay of treatment, yet only a small subset (5% to 15%) of patients with isolated disease qualify for surgical resection.

Because surgery can only remove visible nodules, micrometastatic disease, which makes up a significant proportion of the metastatic burden, is inadequately targeted, driving recurrence rates. Chemotherapy is commonly administered as an adjuvant treatment but fails to control or eradicate the occult metastatic burden. Several factors have been postulated to explain the poor response to chemotherapy, including aggressive tumor biology, the development of chemotherapy-resistant clones of tumor cells, and inadequate chemotherapy doses limited by systemic toxicities.^4^ Even in the setting of optimal treatment, long-term survival is limited, with a 5-year overall survival of 20% to 40% for sarcoma and 54% to 68% for colorectal cancer patients.^2^^,^^3^^,^^5^^,^^6^ Accordingly, alternative treatments are needed to effectively eradicate micrometastatic disease across large tissue volumes without the possibility of selecting therapy resistant clones, leading to treatment failure.

### 1.1 Photodynamic Therapy and Dosimetry

Photodynamic therapy (PDT) is a promising cancer treatment modality that is less susceptible to the genetic expression of tumors and can avoid treatment resistance. PDT is based on the administration of a photosensitive drug which, upon absorption of light of a specific wavelength, generates cytotoxic reactive oxygen species, particularly singlet oxygen, leading to cell death when the photosensitizer-light product exceeds a particular minimum or threshold. PDT was used clinically as early as the 1980s, albeit some limitations have prevented it from gaining widespread use in oncology.^7^^–^^9^ Initial photosensitizers have poor tumor selectivity and long retention times in proliferating tissues, rendering patients sensitive to sunlight for several days to weeks. Given the high attenuation of the photons activating the photosensitizers, particularly by hemoglobin and cytochromes, and the increased path length due to high photon scattering by the lung’s connective tissue and frequent air-tissue interfaces, light penetration in the lung is poor. Hence, PDT was used only for small, early-stage tumors, accessible by bronchoscope or as a palliative treatment to relieve obstruction.^10^^–^^12^

Newer photosensitizers and photosensitizing precursors such as Chlorins, marketed as Temoporfin or Foscan in Europe, and 5-aminolevulinic acid (5-ALA), and others,^10^^,^^13^^,^^14^ respectively, improve tumor selectivity versus normal cells, while reducing the time for skin photosensitivity. Selective uptake and retention of the photosensitizer in tumors results in higher singlet oxygen concentration in the tumor relative to surrounding healthy tissue, eradicating it while limiting collateral damage.

Another development is advances in treatment planning and dosimetry. Akin to radiation oncology, titrating treatment effects to correctly predict the target destruction while minimizing toxicity to healthy tissue is essential. For effective treatment in well-oxygenated tissues such as the lung, including their micrometastases, the PDT dose must exceed a critical threshold in the tumor tissue while not exceeding it in the normal lung.^15^^–^^17^

The threshold value considers a tissue’s ability to mitigate the cytotoxic burden. It is given by its photosensitizer concentration [PS], molar extinction coefficient, ε, and the optical fluence or photon density, Φ, according to T=ε[PS]Φ.^18^^,^^19^ The fluence must be adjusted to meet the required threshold conditions throughout the clinical target volume for a given (PS) in the tumor and the normal host tissue.

### 1.2 Lung Perfusion

Isolated lung perfusion techniques were conceived to deliver treatment to the lung without risk of systemic toxicity.^20^^–^^22^ Based on the Toronto “ex vivo lung perfusion,” a version of isolated “in vivo lung perfusion” (IVLP) was developed employing the identical circuit and perfusion technique to provide a repeatable and non-injurious lung platform, whereby it causes no long-term reduction in lung function. The safety of this technique was demonstrated in pre-clinical acute and survival porcine models for the delivery of chemotherapy.^23^^–^^25^ IVLP drains blood from the lung, resulting in minimal cellular and hemoglobin materials in the lung vasculature after replacement with a low cellular perfusion solution, STEEN (XViVO, Göteborg, Sweden), overcoming one of the light penetration limitations. As shown in our previous work, the low-cellular perfusate results in a significant reduction in the effective attenuation coefficient driven mostly by a decrease in the absorption coefficient. This translated to a 3.3-mm increase in the light penetration depth, equivalent to a three-fold increase in the potential treatment volumes when using a 2-cm long cylindrical diffuser.^26^ IVLP also provides surgical access to the chest cavity for light delivery and allows control of the lung treatment environment (e.g., temperature, pH, drug concentrations, cointerventions).

### 1.3 Rationale and Aims

Two key parameters necessary for lung PDT treatment planning, especially in the context of lung perfusion, have been previously identified.^26^ The first parameter is the optical properties of the lung during both STEEN and blood perfusion, including the effective attenuation, absorption, and reduced scattering coefficients at three wavelengths relevant to the activation spectra of different photosensitizers. The second parameter is the photodynamic threshold of 5-ALA-induced protoporphyrin IX (PpIX) or Chlorin e6-mediated PDT of normal porcine lung tissue.^26^

This study aims to develop a protocol for whole-lung perfusion-assisted PDT with an adequate safety profile and high therapeutic potential. This requires developing and evaluating a light delivery system for homogenous illumination of the entire lung to the minimum required fluence ($J/{\mathrm{cm}}^{2}$) across the treatment planning volume, here ideally an entire lung.

## 2 Materials and Methods

### 2.1 Animals

All animals received humane care as per the Principles of Laboratory Animal Care formulated by the National Society for Medical Research and the Guide for the Care of Laboratory Animals. The study protocol was approved by the Animal Care Research Committee at the Toronto General Hospital Research Institute. A total of 10 Yorkshire male pigs weighing 35 to 40 kg were used in this study.

### 2.2 Testing the Performance of the Light-Emitting Diode Light Delivery System

For this experiment, three Yorkshire male pigs weighing 35 to 40 kg were used and underwent a simplified version of a previously described porcine IVLP survival model.^25^ Briefly, a left thoracotomy was performed with dissection of the left pulmonary artery, left pulmonary vein, and left atrial cuff. After IV administration of 5000 IU of heparin, the left pulmonary artery and atrial cuff were clamped. Following cannulation of the pulmonary artery, a venotomy was created in the left atrium between the left upper and lower pulmonary veins, and the lung was flushed anterograde through the pulmonary artery cannula with 0.5 to 0.75 L of Perfadex, low potassium dextran solution (XVIVO, Goteborg, Sweden).

**Fig. 1:**
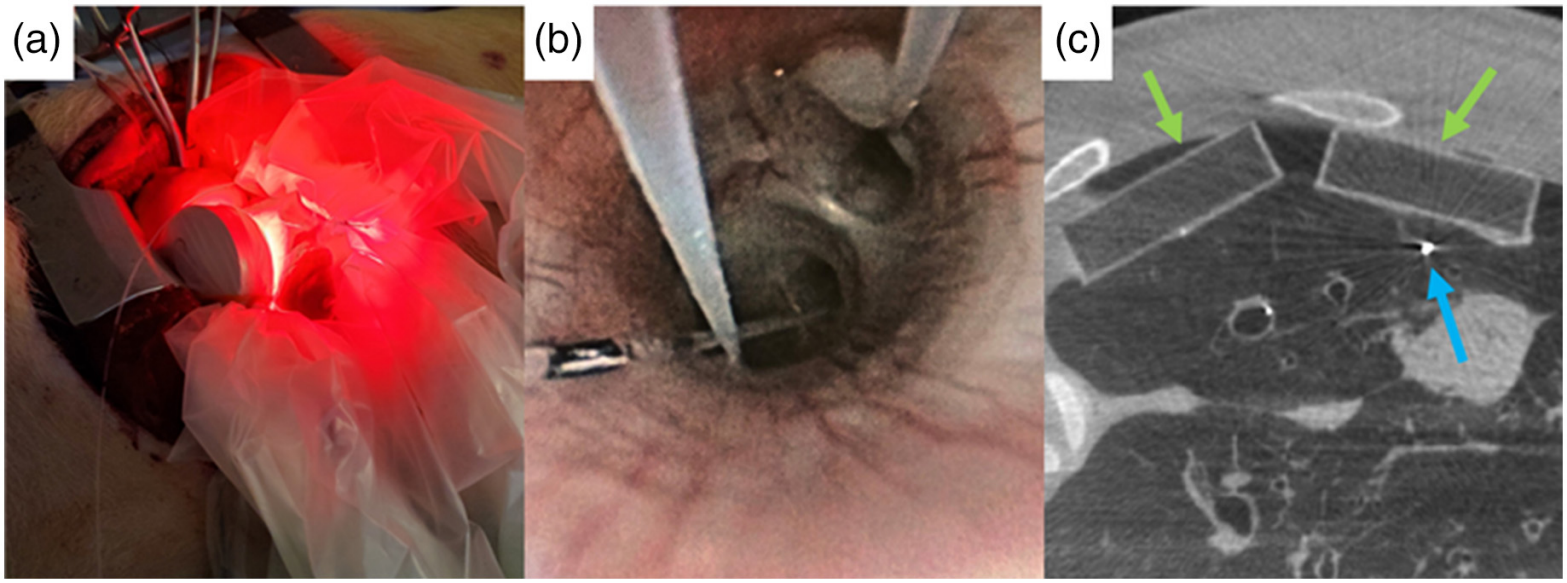
(a) Experimental setup showing light sources wrapped in sterile covers placed within the open chest cavity after lung flush. (b) Bronchoscopic view of isotropic fibers placed within different lung segments. (c) Computed tomography (CT) image of the lung demonstrating the position of the light source phantoms (green arrows) and isotropic fibers (blue arrows).

After the lung flush, up to four custom-built, 5-cm diameter and 1.5-cm thick cylindrical, light-emitting diode (LED) discs, emitting 630 or 660 nm light were placed into the thorax in transparent ultrasound covers (CIVCO, Coralville, Iowa) to maintain sterility [Fig. 1(a)]. Each LED’s emission was quantified at five positions before placement using an externally calibrated fiberoptic probe (IP85 isotropic probe, Écublens, Switzerland) with coupled power meters (PM130D, Thorlabs, Thorlabs Canada ULC, Saint-Laurent, QC, Canada). The inter- and intra-source light fluence rate was 9.87±0.19 and $10.24\pm 0.37\;\mathrm{mW}/{\mathrm{cm}}^{2}$ at 630 and 660 nm, respectively. Two light sources were placed on each of the upper and lower lobes of the lung. Three calibrated isotropic optical fiber detectors (IP85, Medlight, Switzerland) for local fluence rate quantification were placed endobronchially using a bronchoscope (Olympus Medical Canada, Richmond Hill, Ontario, Canada) to ensure the detectors were guided into separate lung segments [Fig. 1(b)]. These fluence-rate detectors were placed deep within the lung past the third order bronchi, and four isotropic fibers were placed between the light source and the lung surface to monitor the sources’ irradiance. The fluence-rate detectors were connected to an in-house built eight-channel LabVIEW-controlled photodiode detector system.^27^ The fluence rate sensor responsivity was corrected for the change of the detectors’ surrounding medium refractive index as required.^28^

A CT scan was performed to provide the spatial relationship between isotropic fibers and light sources [Fig. 1(c)]. As the light sources have metal components, 3D-printed identically sized hollow phantoms replaced the sources for imaging. After the experiment, animals were exsanguinated under anesthesia.

To create an *in silico* model visualizing the fluence-rate distribution in the lung, based on the CT scan, the thorax was segmented into regions comprising light sources, optical fiber detectors, the bronchial tree, lung parenchyma, and bone. These regions were rendered into volumes represented tetrahedrally, thus creating the *in silico* model to visualize the fluence-rate distribution in the lung. The bony thorax had an insignificant effect on the fluence rate distribution and was excluded to reduce the model’s file size. The fluence rate distribution inside the lung was modeled by Monte-Carlo using FullMonte using the tissue optical properties for the various optical components as listed in Table 1.^26^^,^^31^ The validity of the simulation model has been previously described in various contexts, including the lung.^31^^–^^34^

**Table 1:** Optical properties used for the FullMonte-based light propagation simulations.^26^^,^^29^^,^^30^

Material	Scattering coefficient (1/mm)	Absorption coefficient (1/mm)	Anisotropy	Refractive index
Air	0	0	1	1
Source	10	0.001	1	1
Lung	0.965	0.0473	0.93	1.36
Absorber	0.1	$1\times 10^{8}$	0	1
Bone	1.58	0.014	0	1.56
Muscle	7.356	0.052	0.93	1.37

Simulations were performed for all sources independently and with all sources together. The fluence rate measured by the isotropic detector fibers was plotted as a function of the distance to each source and compared with the *in silico* simulation results to confirm the optical properties and the resulting fluence rate gradient. For experiment 1, the simulated and the measured fluence rates were also plotted against each other for comparison. Fluence rate histograms of the pig lung volume were created to estimate the effective lung volume targetable by this illumination approach.

### 2.3 Photodynamic Therapy Protocol Safety and Feasibility Assessment

Seven Yorkshire male pigs weighing 35 to 40 kg were used, four were administered 5-ALA (Sigma-Aldrich, Mississauga, Ontario, Canada), and three were administered Chlorin e6 (Synverdis GmbH, Heidelberg, Germany).

The study used an accelerated dose-escalation study design, whereby the dose of PDT was doubled with each sequential case if no PDT-related toxicity was observed during the previous case.^35^ If the starting dose was not tolerated, the subsequent case used half the previous photosensitizer dose. For the first two cases with 5-ALA and the first with Chlorin e6, different radiant exposures ($J\;{\mathrm{cm}}^{-2}$) were delivered to the upper and lower lobes by adjusting the irradiation time, providing additional data points for the escalation study. Cases to confirm a maximally tolerated dose level were performed with only one radiant exposure for the upper and lower lobes, respectively, to achieve accurate fluence-volume-histograms.

Lung physiology was assessed during the baseline procedure, at reperfusion, and 72 h post-PDT. Gas exchange was evaluated through arterial blood gas analysis. Airway dynamics were assessed through the measurement of dynamic and static compliance.

Pigs underwent a simplified lung perfusion procedure, as described in Sec. 2.2. Once the lung flush had concluded, the PDT procedure was performed. Photosensitizers were given intravenously apart from the third case with Chlorin e6. Following 5-ALA administration and a 3- to 4-h interval, light exposure was started. The starting 5-ALA dose was 60  mg/kg. The three Chlorin e6 cases were administered 1  mg/kg and had drug light intervals of 1 h, 15 min, and 0 min, respectively. For the third chlorin case with a 0-min drug-light interval, a Chlorin e6 loading dose of 10  mg/0.5  L Perfadex flush, equivalent to 1  mg/kg, was administered to achieve the highest tissue concentration over the entire light illumination time, as a worst-case scenario. Because the half-life of Chlorin e6 in plasma is ∼4.8  min, a continuous flush of 2  mg/L/h of Chlorin e6 was maintained during the PDT illumination time.^36^

For the four cases administered 5-ALA and the first case using Chlorin e6, two LED sources, wrapped in transparent ultrasound covers, were placed on the upper and lower lobes, respectively. Isotropic detectors were secured atop the light source to monitor the irradiance. Additional isotropic detectors were placed on the lung surface and within the lung under bronchoscopy guidance, as described above in Sec. 2.2. 5-ALA-induced PpIX and Chlorin e6 were activated at 630 nm delivering 6 to $12\;J/{\mathrm{cm}}^{2}$ and 660 nm delivering 7.2 to $14.4\;J/{\mathrm{cm}}^{2}$, respectively.

The second and third Chlorin e6 cases utilized a fiberoptic-coupled modulight laser (665/808 OEM; Modulight Inc., Tampere, Finland) emitting 665 nm radiation with the goal to achieve a significantly higher administered PDT dose. The optical fiber was equipped with a microlens illuminating a 3.5-cm diameter spot on the lung surface. Irradiance was $200\;\mathrm{mW}/{\mathrm{cm}}^{2}$ delivering a total fluence of 240 to $285\;J/{\mathrm{cm}}^{2}$.

At the conclusion of PDT, the pigs were recovered and treated over a 72-h survival period. Pigs underwent a final procedure where endpoint lung toxicity assessments were made prior to euthanasia by exsanguination under anesthesia. Lung toxicity assessment included physiological, gross, CT, and histological examination. A CT scan was performed at 72 h immediately before the final assessment. The lungs were harvested while inflated after sacrificing for assessment of gross appearance.

Tissue biopsies were collected directly before and after PDT from the lung periphery to quantify the photosensitizer concentration. At 72 h, lung biopsies were taken from the upper and lower lobes centered around areas directly abutting the LED sources. Tissue samples were fixed in 10% phosphate-buffered formalin, embedded in paraffin, sectioned, and stained in hematoxylin and eosin for assessment by light microscopy using a pathologic acute lung injury assessment system described previously described.^37^

Toxicity was defined as a clinically significant impairment of lung function with a ${\mathrm{PaO}}_{2}/{\mathrm{FiO}}_{2}$ (P/F) ratio <300  mm Hg, a reduction of compliance in the treated lung below $10\;\mathrm{mL}/\mathrm{cm}\;H_{2}O$ and/or signs of severe acute lung injury on gross, CT, and histological examination.

### 2.4 Photosensitizer Threshold Assessment

The Chlorin e6 and ALA-induced PpIX concentrations were determined from pre- and post-PDT biopsies by tissue solubilization and fluorescence spectroscopy as published.^38^ The depth of necrosis was measured on H&E slides for all cases with visible necrosis. To quantify the PDT threshold value of both photosensitizers for normal lung tissue, the fluence, Φ, at the necrotic boundary, as simulated using the previously determined tissue optical properties and the measured photosensitizer concentration from the tissue biopsies, was utilized. The molar extinction coefficients of PpIX ($5005\;M^{-1}\;{\mathrm{cm}}^{-1}$ at 635 nm) and Chlorin e6 ($60,386\;M^{-1}\;{\mathrm{cm}}^{-1}$ at 660 nm) were taken from the Oregon Medical Laser Center website.^39^ The derived ALA-induced PpIX PDT threshold values were compared against our previously determined threshold values.^26^

## 3 Results

### 3.1 Testing the Light-Emitting Diode Light Delivery System Performance

**Fig. 2:**
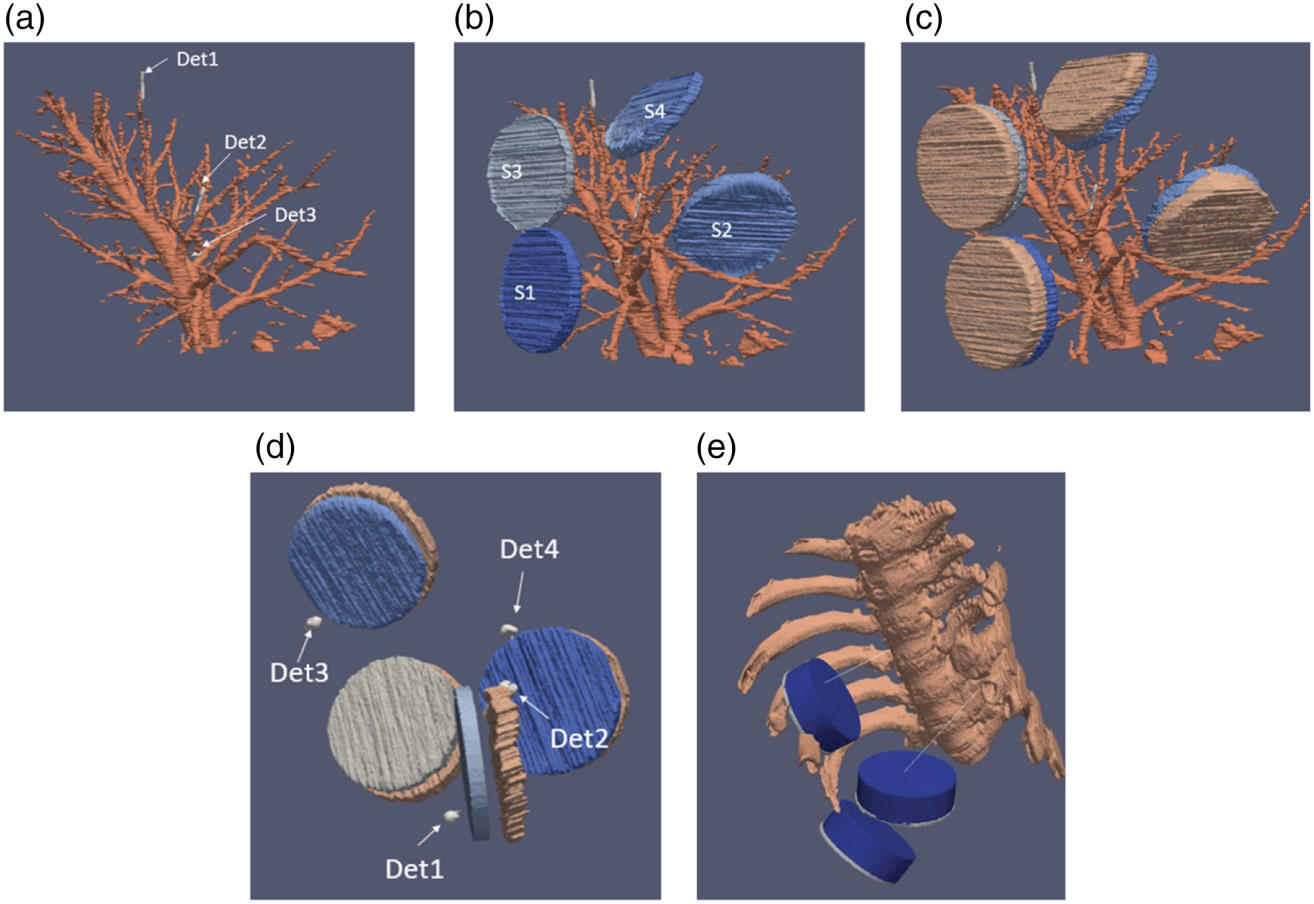
CT scans were segmented for the bronchial tree, lung parenchyma, sources, detectors, and surrounding rib cage. Panels show the progressive layers of the segmentation: (a) the bronchial tree and endobronchial light detectors, (b) the addition of the light sources, (c) the addition of non-reflective and non-emitting back of the light sources, (d) the detectors placed in front of each source as an internal control on max fluence outputted from each source, and (e) position sources in relation to the bony ribcage.

Figure 2 shows segmentation examples of the bronchial tree, lung parenchyma, sources, detectors, and surrounding bony ribcage used in Monte Carlo photon propagation simulations.

**Fig. 3:**
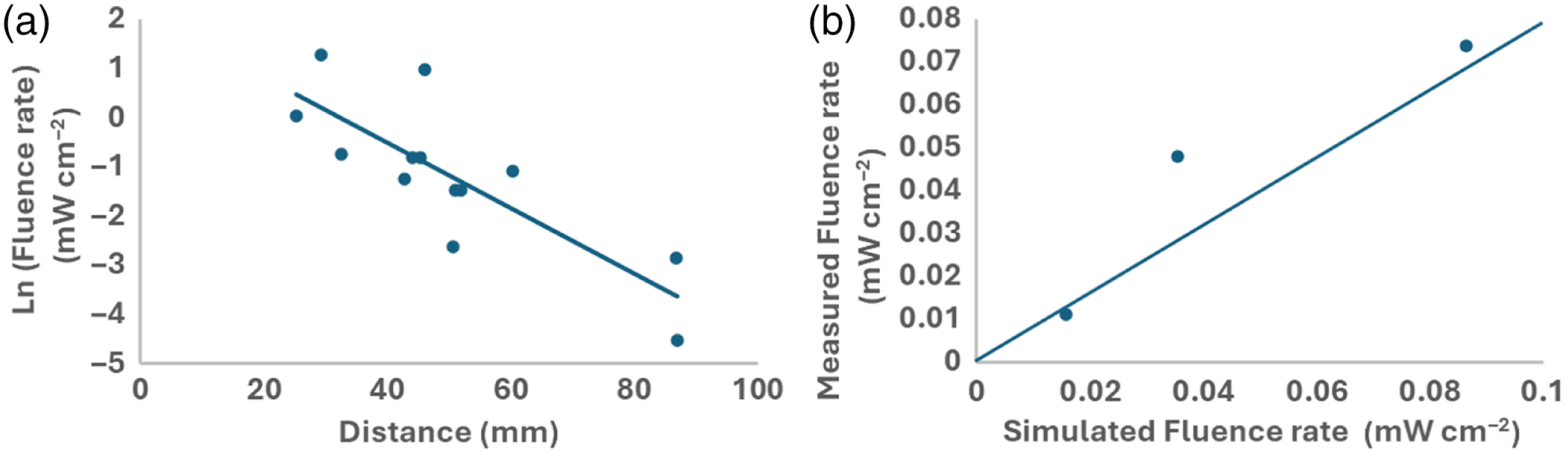
(a) Ln of the fluence rate versus distance to the sources’ center position across four experiments. The slope of the curve provides the effective attenuation coefficient. (b) Comparison of the measured versus the simulated fluence rate for experiment 1.

Figure 3(a) shows the measured fluence rate from sequential illumination by a single source versus a sensor’s relative distance to that source’s center position for the third case of ALA-induced PpIX. This slope provides the experimentally calculated effective attenuation coefficient compared with the lung tissue optical properties, including absorption and scattering coefficients, used in the Monte Carlo simulations. The experimentally derived effective attenuation coefficient was $3.98\;{\mathrm{cm}}^{-1}$ whereas simulated cases used an effective attenuation coefficient of $3.79\;{\mathrm{cm}}^{-1}$ for lung tissue. Figure 3(b) shows the comparison of the simulated and measured fluence rates for one experiment.

Overall, the predicted fluence rate as modeled in the simulation, based on our previously determined optical properties^26^ and compared with the actual measurements from the detectors, shows a strong predictive value of the simulation model toward the fluence rate throughout the clinical target volume.

**Fig. 4:**
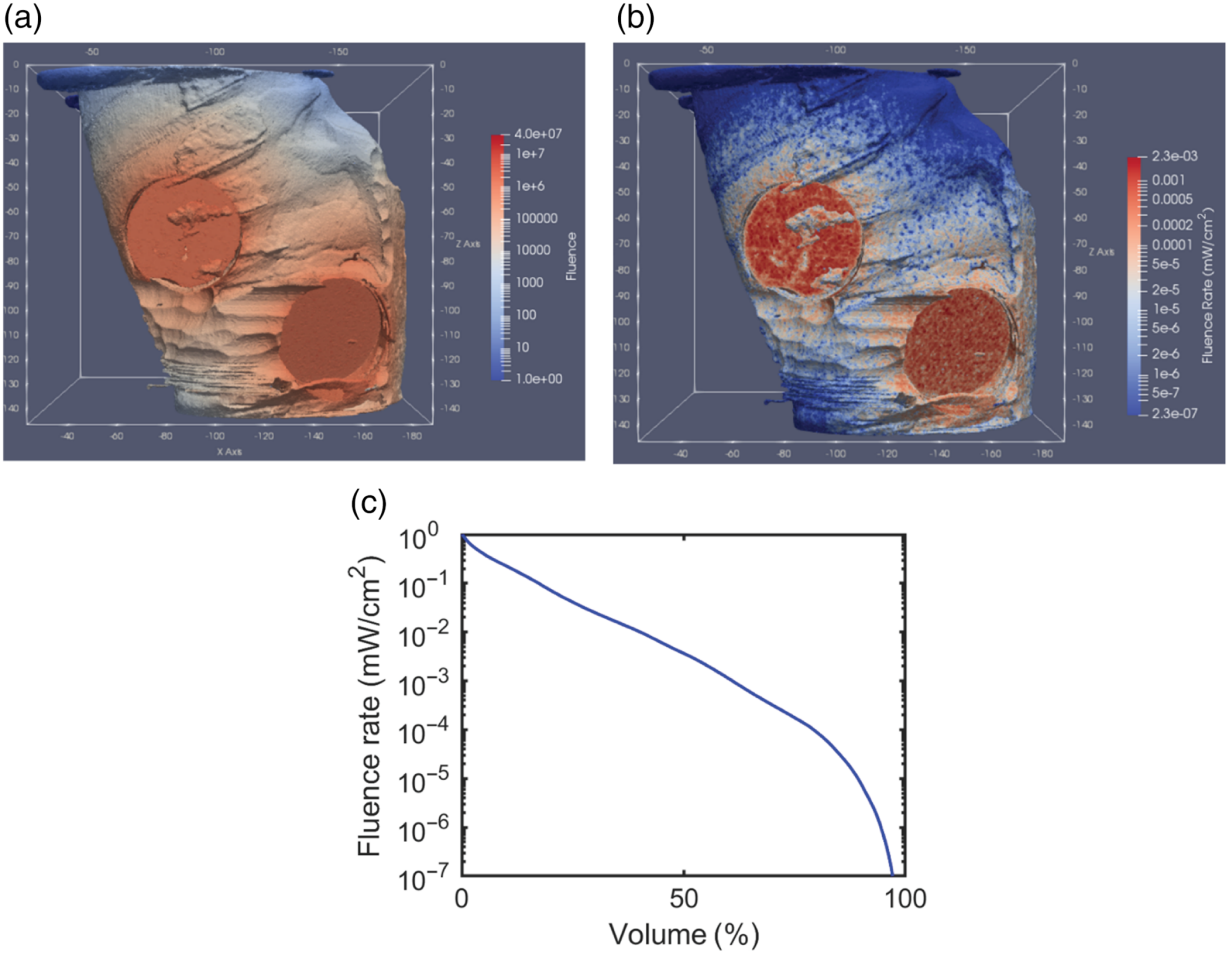
Simulated representations of the dose-volume histogram. (a) Fluence map in units of photon $\text{weight}/{\mathrm{mm}}^{2}$. (b) Fluence rate map in units of $\mathrm{mW}/{\mathrm{cm}}^{2}$. (c) Dose-volume histogram, showing the fluence rate as a function of the lung volume percentage for one case given ALA-induced PpIX.

To determine the attainable fluence rate distribution in the lung based on three 5-cm diameter sources operated simultaneously, dose-volume histograms based on the simulations model demonstrating the light fluence throughout the entire organ (Fig. 4). This figure shows the fluence rate for a lung irradiated with three LED sources emitting 665 nm photons with a total power of 2.43 W. The variation of the fluence rate was within two orders of magnitude over ∼50% of the lung volume despite covering less than 15% of the lung surface by the three light sources.

**Fig. 5:**
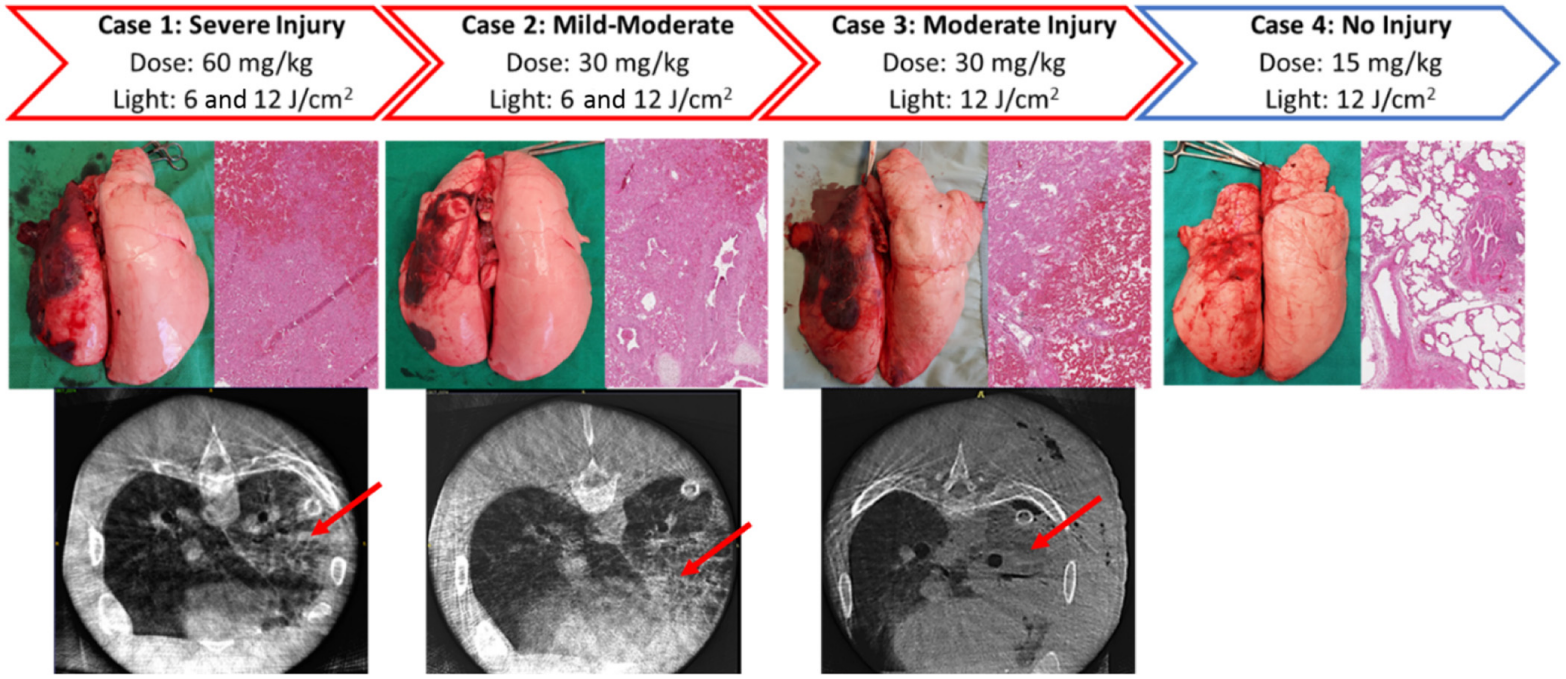
An overview of cases administered ALA. (Top row) Case numbers with ALA and light doses administered, the drug-light interval for PDT treatments, and the resulting degree of lung injury. (Middle row) Gross and histological appearance of lungs at 72 h. (Bottom row) CT images at 72 h, red arrows indicating areas of consolidation. The fourth case did not undergo CT scanning. Only the fourth case showed an undamaged gross and histological appearance.

The dose-volume histograms from simulations and our direct measurements consistently demonstrated fluence rates $>1\;\mathrm{mW}/{\mathrm{cm}}^{2}$ and $>10\;\mu W/{\mathrm{cm}}^{2}$ across ∼25% and 80% of a single lung, respectively, comprising the clinical target volume.

### 3.2 Photodynamic Therapy Protocol Safety and Feasibility Assessment

All animals tolerated the procedure well, with no adverse events from the surgical procedure. All cases demonstrated preserved lung function and physiology at the end of the PDT treatment with a stable P/F ratio above 300 mmHg, preserved lung compliance, and no signs of significant lung injury on gross or histological appearance. All animals survived to the 72 h endpoint in good clinical condition.

#### 3.2.1 ALA dose de-escalation

**Fig. 6:**
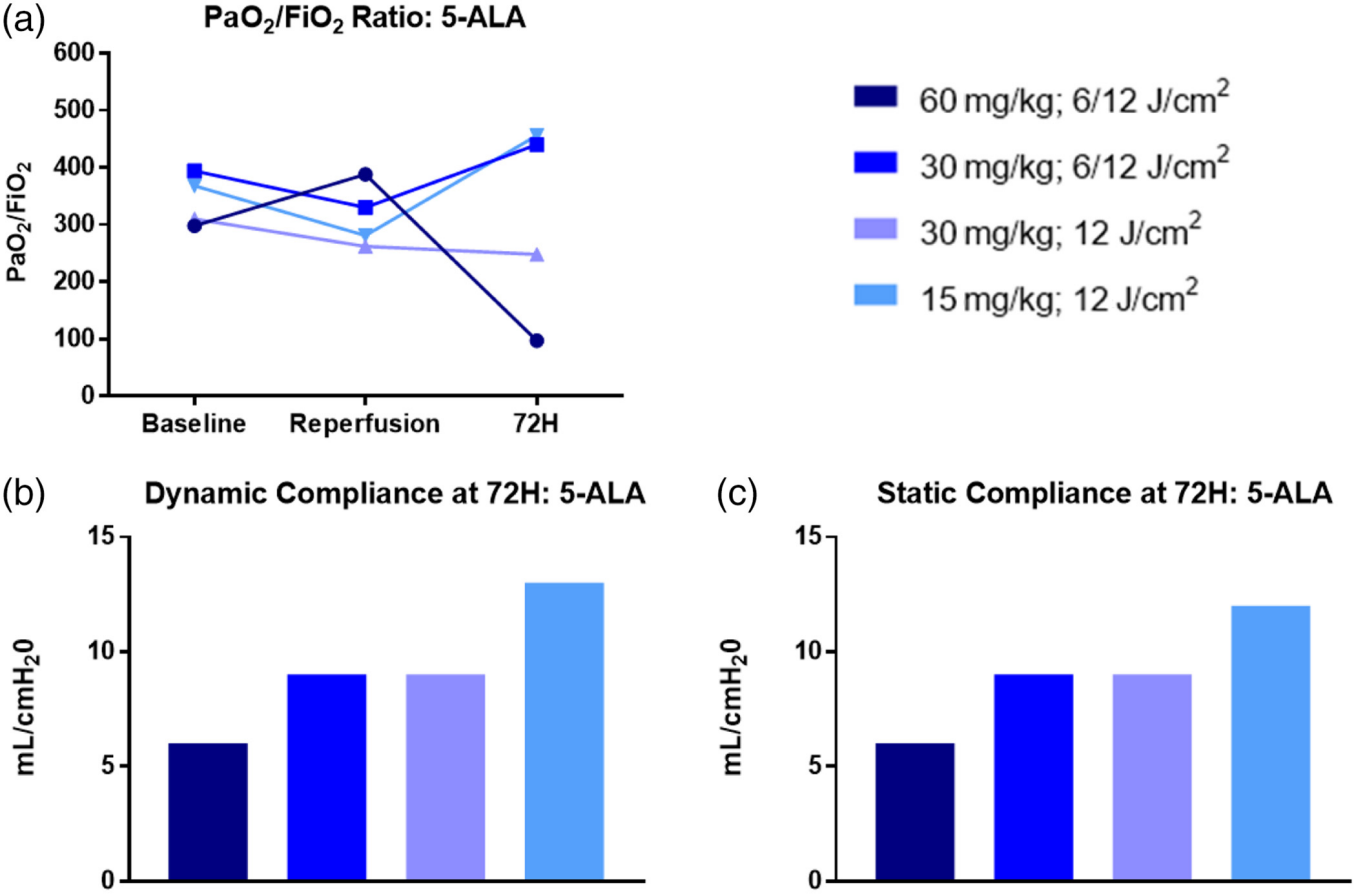
(a) ${\mathrm{PaO}}_{2}/{\mathrm{FiO}}_{2}$ ratio over the 72-h experimental timeframe. A ratio below 300 was considered clinically significant. (b) Dynamic compliance of the treated left lung at 72 h. (c) Static compliance of the treated left lung at 72 h. Only the fourth case demonstrated preservation of the ${\mathrm{PaO}}_{2}/{\mathrm{FiO}}_{2}$ ratio and dynamic and static compliance.

An overview of the cases administered ALA is displayed in Fig. 5. Lung physiological assessments for the ALA cases are shown in Fig. 6.

**Fig. 7:**
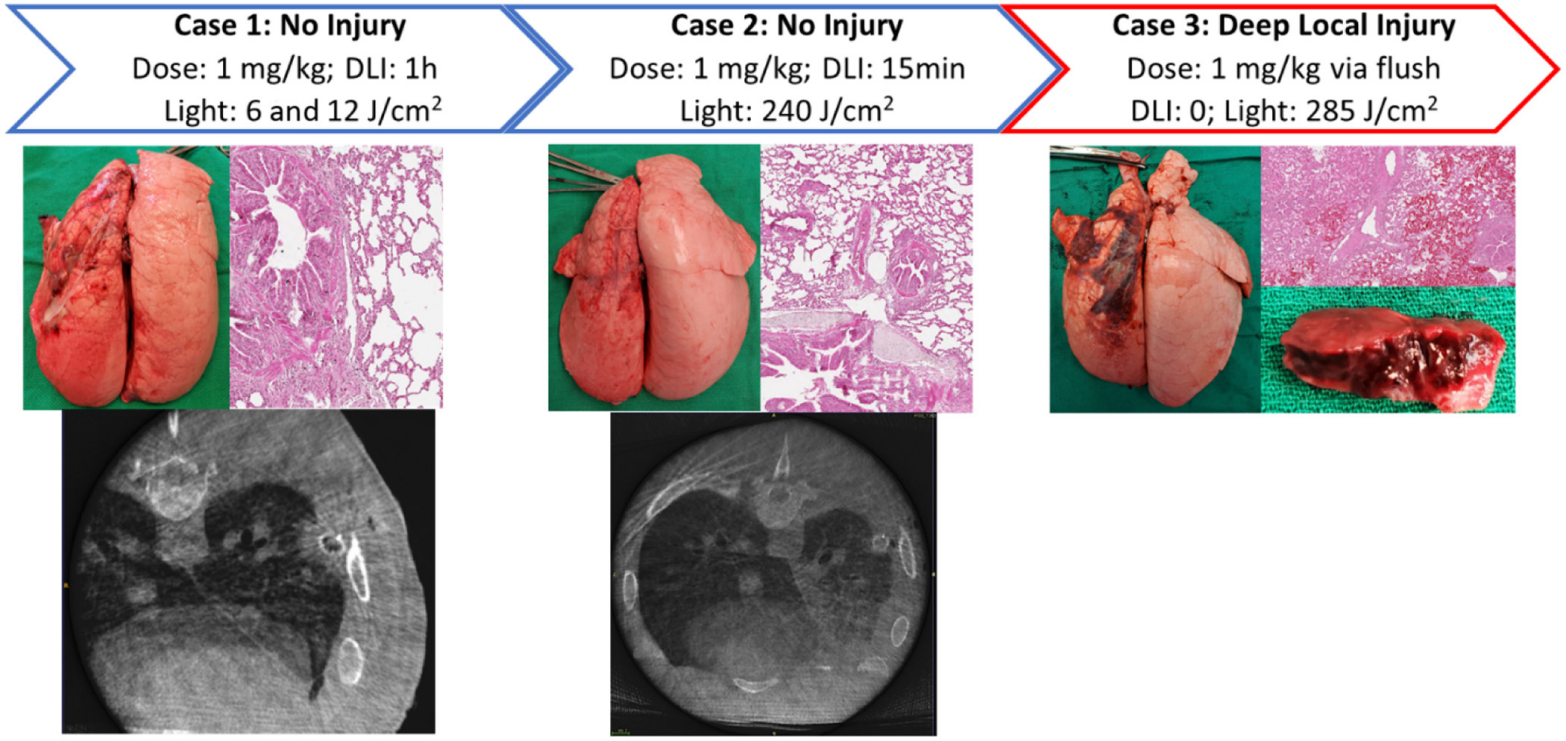
An overview of cases administered Chlorin e6. (Top row) Case numbers with Chlorin e6 and light doses administered, the drug-light interval for PDT treatments, and the resulting degree of lung injury. (Middle row) Gross and histological appearance of lungs at 72 h. (Bottom row) CT images of lungs at 72 h. The third case did not undergo CT scanning.

The first case using an ALA dose of 60  mg/kg demonstrated severe lung injury with a P/F ratio of 97, dynamic and static compliance of $6\;\mathrm{mL}/\mathrm{cm}\;H_{2}O$, signs of necrosis and severe inflammation on both gross and histological examinations. Injury in the upper lobe, irradiated at $12\;J\;{\mathrm{cm}}^{-2}$, was greater than for the lower lobe, irradiated at $6\;J/{\mathrm{cm}}^{2}$, as shown by a P/F ratio of 46 versus 189 from the left upper and lower pulmonary veins, respectively. The ALA dose was halved for case 2 and yielded a milder but still clinically significant injury to the normal lung with a P/F ratio of 440, dynamic and static compliance of $9\;\mathrm{mL}/\mathrm{cm}\;H_{2}O$, and a shallower necrosis and lung injury pattern on gross, CT, and histological examination. There was no noticeable difference in the injury extent between the upper and lower lobes irradiated at 12 and $6\;J/{\mathrm{cm}}^{2}$, respectively. Case 3 was repeated at $30\;\mathrm{mg}\;{\mathrm{kg}}^{-1}$ but with a single light dose of $12\;J/{\mathrm{cm}}^{2}$ to confirm toxicity at this optical dose, given case 2 had borderline clinically relevant lung injury. This case showed a more moderate lung injury with a P/F ratio of 248, dynamic and static compliance of $9\;\mathrm{mL}/\mathrm{cm}\;H_{2}O$, and a more profound degree of necrosis and inflammation on gross, CT, and histological appearance. The ALA dose was lowered to 15  mg/kg for case 4. This case demonstrated no lung injury with a P/F ratio of 456, dynamic and static compliance of 13 and $12\;\mathrm{mL}/\mathrm{cm}\;H_{2}O$, respectively, and no evidence of necrosis or inflammation on gross or histological examination.

#### 3.2.2 Chlorin e6 dose escalation

**Fig. 8:**
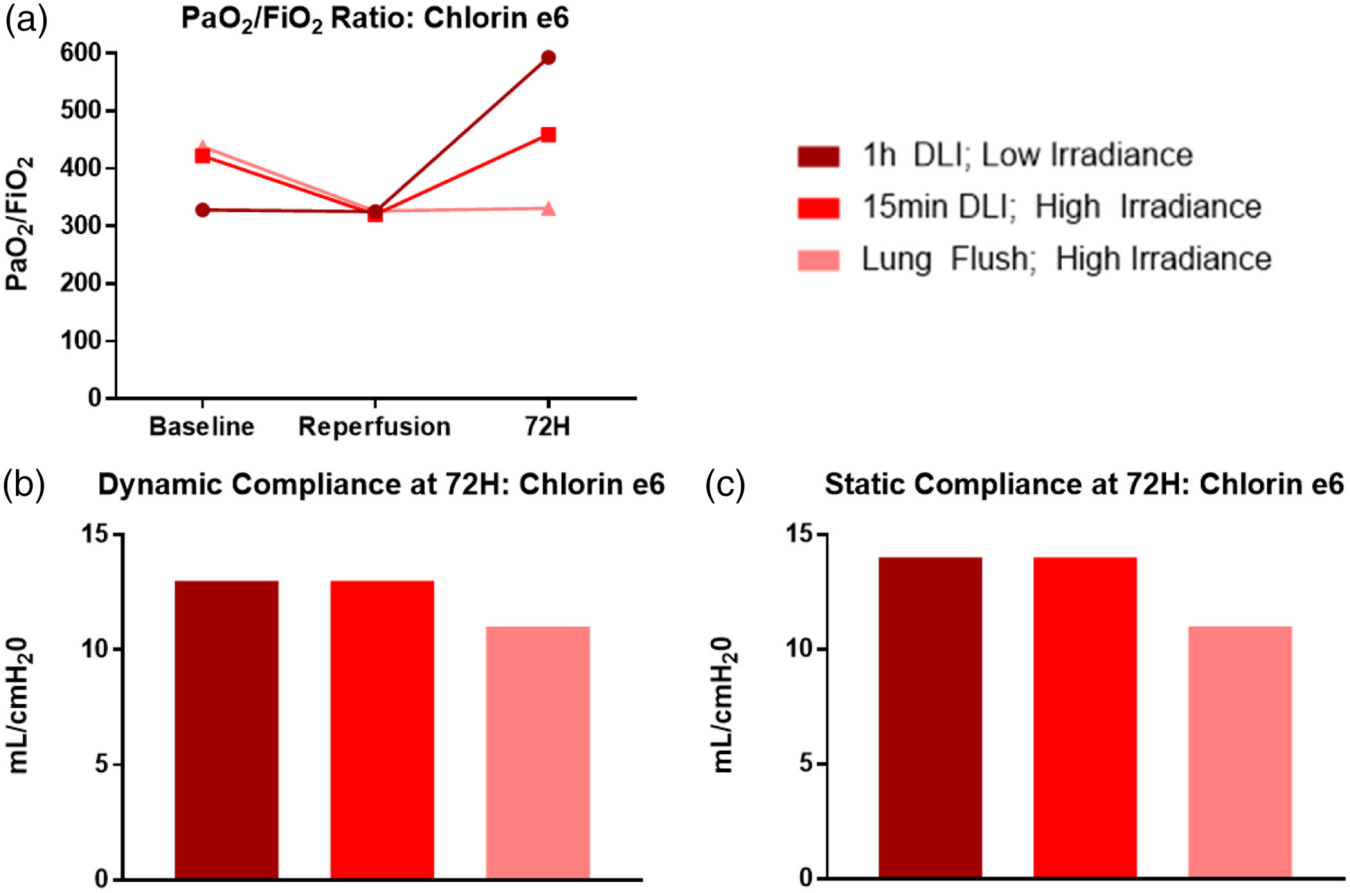
(a) ${\mathrm{PaO}}_{2}/{\mathrm{FiO}}_{2}$ ratio over the 72 h experimental timeframe. A ratio below 300 was considered clinically significant. (b) Dynamic compliance of the treated left lung at 72 h. (c) Static compliance of the treated left lung at 72 h. Only the third case demonstrated mild lung injury.

Figure 7 provides an overview of the cases administered Chlorin e6 and Fig. 8 shows lung physiological assessments for the Chlorin e6 cases.

**Fig. 9:**
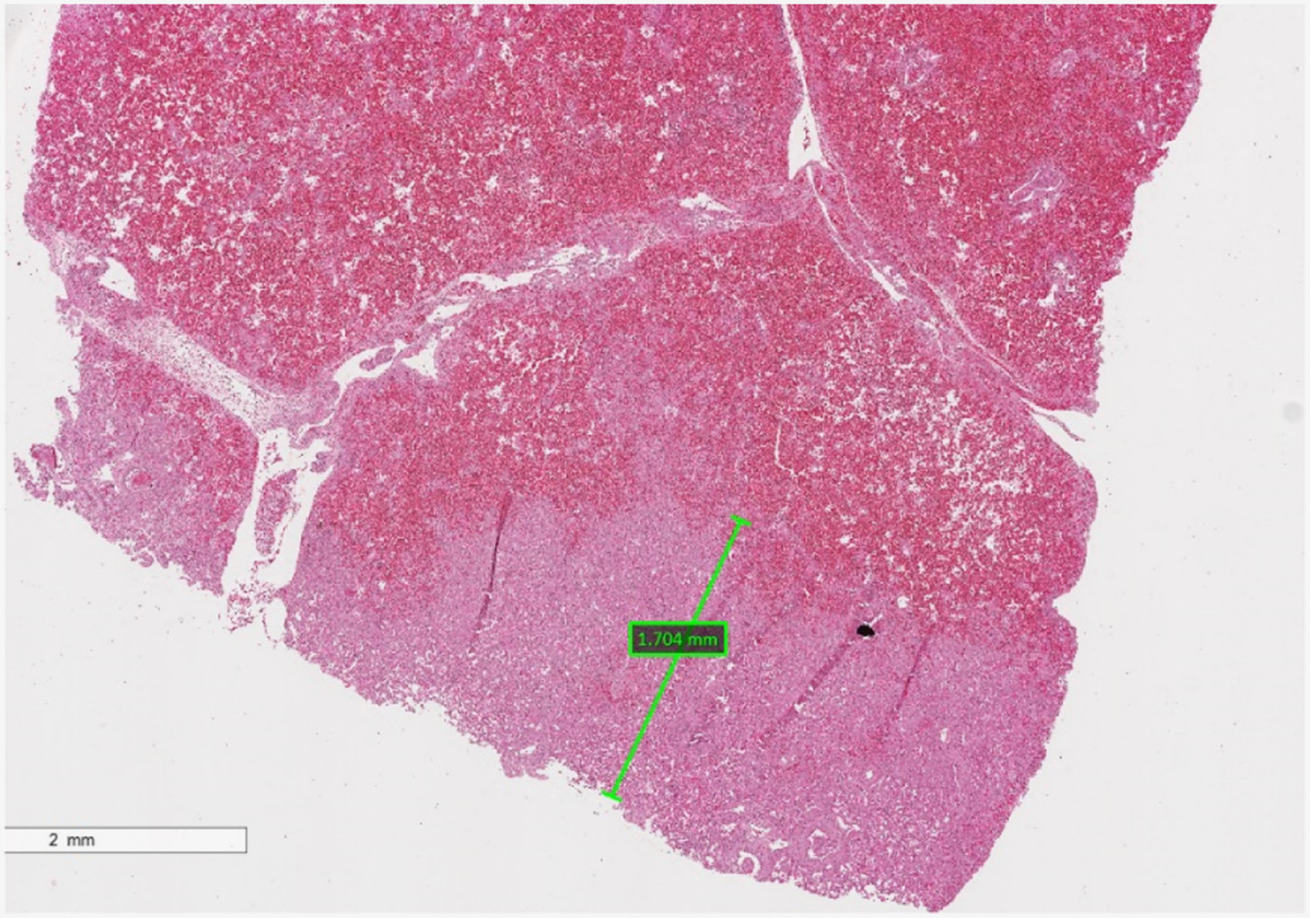
Histology with H&E staining of an area of necrosis. Maximum depth of necrosis was measured, as shown with the green bar. Surrounding tissue showed signs of severe inflammation including patchy necrosis, fibrin deposition, and airspace hemorrhage.

The first Chlorin e6 case delivered PDT comprised $1\;\mathrm{mg}\;{\mathrm{kg}}^{-1}$ and a radiant exposure of 12 and $6\;J/{\mathrm{cm}}^{2}$ for the upper and lower lobes, respectively, resulting in no evidence of necrosis or inflammation on gross, CT, or histological examination. The P/F ratio remained normal at 593, and dynamic and static compliance were 13 and $14\;\mathrm{mL}/H_{2}O$, respectively. Given the lack of demonstratable PDT effect and our previous study showing no Chlorin e6 uptake in healthy tissue after a 1-h drug-light interval,^26^ an increase in drug dose far beyond clinical protocols may be required to produce a PDT effect in normal tissue. The subsequent two cases were performed to elucidate the minimum PDT threshold or maximally tolerated dose in normal lung tissue rather than a strict dose-escalation design. Case 2 was performed with a radiant exposure of $240\;J/{\mathrm{cm}}^{2}$ via irradiation of a 5- to 6-cm diameter spot by collimated laser light to cause local necrosis. A shorter drug-light interval of 15 min was employed to retain some photosensitizer in the normal lung tissue. Again, no evidence of necrosis or inflammation on gross, CT, or histological examination was noted, with a P/F ratio of 459 and dynamic and static compliance of 13 and $14\;\mathrm{mL}/\mathrm{cm}\;H_{2}O$, respectively. To further minimize the drug-light interval, the final case was performed with Chlorin e6 administered through the lung flush continuously during the delivery of $285\;J/{\mathrm{cm}}^{2}$ radiant exposure, whereby the photosensitizer acted on the tissue and the vasculature. This resulted in deep local lung injury over the irradiated area with a P/F ratio of 331 and dynamic and static compliance of $11\;\mathrm{mL}/\mathrm{cm}\;H_{2}O$. Necrosis extended up to 11 mm into the irradiated lung.

#### 3.2.3 Photosensitizer threshold assessment

Histologic assessment of the depth of necrosis (Fig. 9) was used to establish the photodynamic threshold values for both photosensitizers, listed in Table 2. The necrosis threshold values for ALA-induced PpIX based on all data sets was $2.10\times 10^{17}\pm 8.24\times 10^{16}\;h\nu /{\mathrm{cm}}^{3}$. Given that the Chlorin e6 lung tissue concentrations were below the detection limit for the first two cases, it was impossible to calculate the photodynamic threshold of Chlorin e6 following i.v. administration. Based on the third case of Chlorin e6, the minimum threshold can be estimated to be at least $6.30\times 10^{15}\pm 8.48\times 10^{15}\;h\nu /{\mathrm{cm}}^{3}$ when using Chlorin e6 as a vascular photosensitizer.

**Table 2:** Case-based factors measured for photodynamic threshold determination, including light dose delivered, tissue photosensitizer concentration, and depth of necrosis.

PS/case #	PS dose (mg/kg)	Light dose ($J/{\mathrm{cm}}^{2}$)	PS [C] (μg/g)	PS [C] (μM)	Depth of necrosis (mm)	Threshold, T ($h\nu /{\mathrm{cm}}^{3}$)
ALA/1	60	6/12	0.96 ± 0.71	57.2	1.0/1.7	$2.10\times 10^{17}\pm 8.24\times 10^{16}$
ALA/2	30	6/12	0.062 ± 0.05	28.6	0.7/1.4	$1.85\times 10^{17}\pm 5.36\times 10^{16}$
ALA/3	30	12	0.052 ± 0.06	28.6	1.0–1.2	$1.12\times 10^{17}\pm 1.24\times 10^{17}$
ALA/4	15	12	0.032 ± 0.23	14.3	0	N/A
Chlorin/1	1	7.2/14.4	<0.01	N/A	0	N/A
Chlorin/2	1	240	<0.01	N/A	0	N/A
Chlorin/3	1	285	2.2 ± 1.76	503	11	$6.30\times 10^{15}\pm 8.48\times 10^{15}$

For ALA cases 1 and 2, the two depths of necrosis presented correspond to the two light doses administered.

## 4 Discussion

This technique has several important implications when considering PDT treatments in the lung. The delivery of a selective PDT protocol requires accurate dosimetry that can be used to predict a desired treatment effect. Tissue response to PDT can be described by a threshold concept, which is defined as the optical fluence and tissue photosensitizer concentration required to result in PDT-induced necrosis of the target tissue.^19^ Tumor tissues often demonstrate a higher PDT threshold than cancer cells due to differences in their resilience from oxidative stress. Conversely, many photosensitizers work by exploiting increased accumulation and retention of photosensitizers in cancer cells compared with normal cells.^13^ These phenomena need to be balanced in favor of the host tissue to allow for a potential therapeutic window for PDT treatment above the threshold of the cancer cells but below that of normal tissue across the entire lung volume.

The creation of a whole-lung PDT procedure targeting lung metastases has not been previously reported. The challenge is providing a sufficient fluence rate across the entire organ while maintaining sufficient selectivity of the PDT effect toward the metastases to spare healthy tissue. Herein, a path toward overcoming these challenges and delivering whole-lung PDT for diffuse metastatic disease is presented. A light delivery system for improved illumination of the lung with increased treatment volumes comprised of multiple cylindrical LED light sources at the desired light wavelengths, here 630 or 660 nm, is presented. The light source aperture area comprises up to $314\;{\mathrm{cm}}^{2}$ and can deliver up to 3.235 W optical power.

Our previous work compared the optical properties of the lung during normal blood perfusion conditions with perfusion using an acellular perfusion solution. Acellular perfusion resulted in significant reduction in the tissue absorption coefficient resulting in a 3.3-mm increased penetration depth of light and accordingly a three-fold increase in effective treatment volumes.^26^ Using these previously determined optical properties for the lung tissue as input parameters for Monte Carlo simulations of the fluence rate throughout the lung, based on the source positions obtained from CT imaging. The source intensities are subject to assumption of homogeneous, Lambertian emission. The lung tissue was assumed to be a turbid homogenous medium beyond the 8th generation airways as resolving for smaller airways has no significant effect on fluence rate calculations.^40^ Up to the 8th generation, the initial bronchial tree was segmented as empty air spaces. Comparison of the simulated fluence rate and effective attenuation with direct measurements taken during the cases exhibited high concordance, as shown in Fig. 3, validating this technique to generate dose-volume histograms.

While there is a significant increase in the effective treatment volume of the lung, a further expansion of the light-emitting surface area is required, as at present <20% of the surface area is directly illuminated. Other groups have previously developed flexible radiation-emitting devices that conform to complex surfaces, such as the Freiburg Flap^41^ or light-emitting textiles.^42^^,^^43^ The former, initially developed for brachytherapy application, can be outfitted with optical fibers in place of the radiation sources, to provide reliable light intra-operatively to the lung that is amenable to dosimetry planning.^44^ However, both techniques emit light toward the lung and the pleural cavity, and additional reflective materials must be placed between the light source and the pleural wall.

A limitation of the LED device presented here may be the low attainable fluence rates of 1 to $3\;\mathrm{mW}/{\mathrm{cm}}^{2}$, which are significantly below 50 to $150\;\mathrm{mW}/{\mathrm{cm}}^{2}$ typically employed in PDT protocols, leading to long irradiation times in the operating room, and increased power output without causing thermal heating is necessary. The required fluence rate or photodynamic threshold for PDT of occult micrometastatic disease is currently unknown. Previous clinical protocols have delivered high PDT radian exposures ($>100\;J/{\mathrm{cm}}^{2}$) to small target areas containing gross tumors.

The feasibility and safety profile of whole-lung PDT using large-area light delivery devices placed in the thoracic cavity were demonstrated. Photosensitizers are usually given intravenously at an appropriate drug-light interval. Here, a modified lung flush procedure was employed to drain the organ of blood and allow improved light penetration, resulting in a 3.3-mm increased light penetration. Examining the cases administered 5-ALA, the maximally tolerated dose for normal lung tissue was 15  mg/kg of 5-ALA and a radiant exposure of $12\;J/{\mathrm{cm}}^{2}$. ALA-induced PPIX-mediated PDT demonstrated a dose-response relationship dependent on both, the photosensitizer concentration and delivered radiant exposure. The calculated threshold value for a normal porcine lung is $2.10\times 10^{17}\pm 8.24\times 10^{16}\;h\nu /{\mathrm{cm}}^{3}$, representing the upper limit of the therapeutic window, not to be exceeded throughout the clinical target volume. Regarding the Chlorin e6 cases, no PDT effects or photosensitizer uptake was demonstrated in the lung tissue at clinically used drug-light intervals, even after administering highly elevated radiant exposures. In an additional case to estimate the minimum threshold dose, Chlorin e6 was employed as a vascular-acting photosensitizer by delivering it via the lung flush solution while simultaneously illuminating select lung surface areas. As anticipated, this case demonstrated a vigorous PDT response with deep local injury to the lung and a minimum threshold value of $6.30\times 10^{15}\pm 8.48\times 10^{15}\;h\nu /{\mathrm{cm}}^{3}$ lower than other PDT thresholds reported in the literature for cellular-acting photosensitizers. This protocol does not correspond to the usual drug-light interval used for Chlorin e6, which is aimed at targeting the tumor cellular compartment. The low threshold values could be explained as cellular and vascular PDT are performed. The clinical protocol involves a typical 3- to 4-h drug-light interval, based on the longer retention time of Chlorin e6 in tumor cells while clearing mostly from normal cells. Hence, the calculated PDT threshold values represent the upper therapeutic window limit for normal lung tissues. Previous work has demonstrated the selective uptake of Chlorin e6 up to nine-fold greater in tumor cells compared with normal cells, including sarcoma, small cell and non-small cell lung cancers, and nasopharyngeal carcinoma, in various small animal tumor and xenograft models.^45^^–^^47^ Similarly, chlorin e6 delivered by nano-emulsion has demonstrated significantly greater accumulation in lung metastases compared with normal lung tissue in a mouse tumor model.^48^ The significance of our results is that if it demonstrates similar selective uptake in tumor versus healthy lung tissues, Chlorin e6 could be an ideal photosensitizer for whole-lung PDT. Rapid clearing from normal lung tissue permits delivery of high irradiances and radiant exposure doses to maximize metastatic tumor destruction over an entire lung while avoiding normal tissue toxicity.

However, exploiting this therapeutic window requires dosimetry that allows safe and accurate targeting of the lung while sparing other organs or structures at risk within the high fluence field. A light delivery system similar to that demonstrated here, in combination with the patient-specific simulations, can provide the required dosimetric control. Simulations based on CT images providing dose-volume histograms pre-procedure can guide the required number, area, position, and irradiances of sources to achieve the desired PDT treatment effect.

This study has limitations that must be considered when extrapolating the results presented herein. From the light delivery system and simulation perspective, a porcine model was used with adolescent, healthy pigs, which may not accurately represent a human clinical setting. We previously reported the optical properties of human lungs;^26^ however, the validity of these properties for human lungs with different pathologies and baseline characteristics is unknown, and hence, the accuracy of the simulations may be limited. Regarding the potential selectivity and efficacy of our perfusion-assisted PDT protocol, this study did not include a tumor model. Therefore, we cannot confirm that the photosensitizers would effectively kill tumor cells and provide a targetable therapeutic index without significant toxicity. Selectivity is extrapolated from established threshold values for ALA-induced PpIX and Chlorin e6 for various cancers, which are lower than those we have reported here in normal lung tissue.^19^^,^^49^^,^^50^ Hence, *in vivo* studies with metastatic lung tumors are required to demonstrate the efficacy of this protocol. In addition, while this study has demonstrated the safety of this perfusion-assisted PDT protocol for ALA-induced PpIX and Chlorin e6 during a subacute timeframe, long-term safety was not examined. However, PDT-induced necrosis occurs within a 72-h timeframe and is not known to provide late side effects in other settings.^13^ Finally, using a large animal model, this study necessitated a low sample size, and thus, the results are susceptible to significant case-by-case variation. However, as a proof-of-concept study demonstrating a novel potential treatment protocol, determining exact safety parameters was not the objective of this work.

## 5 Conclusion

This study demonstrated a whole-lung perfusion-assisted PDT protocol. A light delivery system for improved PDT illumination homogeneity of the lung was presented. A simulation-based approach to precise PDT dose treatment planning was presented. The use of the light delivery system was demonstrated, identifying the maximally tolerated dose and feasibility of a perfusion-assisted whole-lung PDT protocol with 5-ALA and Chlorin e6. This protocol demonstrated therapeutic potential for both photosensitizers, with Chlorin e6, demonstrating minimal uptake in healthy lung tissue, with the possibility of sufficiently high therapeutic selectivity to target multifocal lung cancer. This work will inform further study confirming the efficacy of this protocol in tumor models and providing the basis for future clinical translation. Furthermore, it outlines an important approach to dosimetry in the lungs and other solid organs that can be used for personalized PDT protocols.

## Data Availability

Software code for Fullmonte is available at https://gitlab.com/FullMonte/FullMonteSW/-/wikis/home.

## Supporting Data

The supporting data will be made available by the corresponding author upon request.
